# Disease knowledge after an educational program in patients with GERD – a randomized controlled trial

**DOI:** 10.1186/1472-6963-8-236

**Published:** 2008-11-13

**Authors:** Jorgen Urnes, Hermod Petersen, Per G Farup

**Affiliations:** 1 Department of Community Medicine and General Practice, Norwegian University of Science and Technology, Trondheim, Norway; 2 Department of Occupational Health, Trondheim University Hospital, Norway; 3 Trondheim University Hospital Abdominal Centre, Norway; 4 Unit for Applied Clinical Research, Norwegian University of Science and Technology, Trondheim, Norway

## Abstract

**Background:**

Patient education has proved beneficial in several but not all chronic disease. Inconsistent findings may rely on varying educational effects of various programs and differential effects on subgroups of patients. Patients' increase in disease knowledge may serve as a feedback to the educator on how well the education program works – but may not be associated to relevant clinical outcomes like quality of life (QoL). This study aimed to investigate the effects of a group based education program for patients with gastroesophageal reflux disease (GERD) on disease knowledge and the association between knowledge and QoL.

**Methods:**

Patients with GERD were randomly allocated to education (102 patients) or control (109 patients). The education program was designed as a structured dialogue conveying information about pathophysiology, pharmacological and non-pharmacological treatment of GERD, patients' rights and use of healthcare. Outcomes were a 24 item knowledge test on GERD (score 0 – 24) 2 and 12 months after the educational program and disease specific and general QoL (Digestive symptoms and disease impact, DSIQ, and General Health Questionnaire, GHQ).

**Results:**

Patients allocated to education achieved higher knowledge test scores than controls at 2 months (17.0 vs. 13.1, p < 0.001) and at 12 months (17.1 vs. 14.0, p < 0.001) follow-up. Knowledge test score was positively associated with having completed advanced school and inversely related to psychiatric illness and poor QoL as perceived by the patients at the time of inclusion. Overall, changes in knowledge test score were not associated with change in QoL.

**Conclusion:**

A group based education program for patients with GERD designed as a structured dialogue increased patients' disease knowledge, which was retained after 1 year. Changes in GERD-knowledge were not associated with change in QoL.

**Trial registration:**

ClinicalTrials.gov: NCT0061850

## Background

Patient education has proved beneficial in several but not all chronic disease [1-8]. In a patient education program on gastroesophageal reflux disease (GERD-education) we found improvement in quality of life (QoL) in patients with primary school only, while patients who had completed advanced school experienced no effect [9]. These inconsistencies may rely on varying educational effects of various methods and contents, as well as on differential effects on subgroups of patients.

Patient education exerts its effect through patient "learning". Only a few studies have been performed to evaluate the effect of patient education on patient learning and the relationship between patient learning and quality of life [10-13]. Learning may relate to knowledge, skills and attitude [14-17]. Knowledge is an easy and readily available proxy measure of learning and may serve as feedback to the educator on how well the education program performs.

In order to improve patient education programs it is necessary to understand what contents and methods are most suitable with regard to patient learning and how this learning relates to relevant clinical endpoints. In this article we report the effect of a dialogue based patient education program on GERD-related knowledge in patients with mild GERD compared to what is achieved in routine care. Secondary to this, we report how increase in GERD-related knowledge was associated with improvement in QoL, and if this relationship was different in patients with primary school only and those who had completed advanced school.

## Methods

### Patients

The study population was patients with mild GERD, living in the vicinity of the hospital, who were to be followed up by their general practitioner after having been to ambulatory gastroscopy. Inclusion criteria were symptoms dominated by heartburn and/or acid regurgitation, to be in need of symptom relief at least 5 days per week when the symptoms were at their worst during the last year, to have a disease history of at least three months, to be between 20 and 75 years and able to give an informed consent to participate. As patients with present or previous esophagitis grade II or more (Savary-Miller classification) tend to be followed up by gastroenterologists at the hospital, they were excluded. Other exclusion criteria were peptic ulcer disease, established continuous need of proton pump inhibitors or NSAIDs, or a wish for surgical treatment. Earlier surgical treatment in the proximal GI-tract, having another disease affecting quality of life, pregnancy, alcoholism or drug abuse or not being competent to participate in an educational program also excluded patients from participation.

### Patient characteristics and questionnaires

Socio-demographic data, disease characteristics and history, and QoL were recorded in a structured inclusion interview before randomisation. Formal education was recorded as having completed primary school only and having completed advanced school (more than 10 years formal education). Prior to the study patients' response to H2-blocker (ranitidine) was tested and they were characterised as *ranitidine responders *or not.

Quality of life was recorded by the *General Health Questionnaire, 30 item version (GHQ-30) *[18] and the *Digestive Symptoms and Impact Questionnaire, DSIQ *[19]. GHQ-30 has 5 subscales: Anxiety, Well-being, Depression, Social dysfunction and Coping. DSIQ has 5 subscales: Reflux, Gastric dysfunction, Pain and bowel symptoms, Health impairment and Life impairment. Both questionnaires were scored on likert scales where low scores indicate better QoL. GHQ-30 likert sum-score (GHQ-lss) was the sum of all item scores. DSIQ sum-score (DSIQ-ss) was the mean of all item scores.

At 2 and 12 months follow-up data corresponding to baseline were collected by postal questionnaires and telephone interviews by research assistants, in addition to the GERD knowledge test.

*The GERD-knowledge test *consisted of 24 statements regarding GERD (appendix). The patients classified the statements as "true" or "false" or declared "don't know". A panel of non-medical and medical staff at our institute tested its comprehensibility. The correct classifications of the statements as "true" or "false" were confirmed by gastroenterologists at the hospital abdominal centre. GERD-knowledge test score (GERD-knowledge) was the sum of correct responses giving a score range from 0 – 24.

### Randomisation

Randomisation was performed in blocks according to scheduled teaching sessions. After being included, the patients were asked whether the dates of the next session suited them. If they answered "yes", they would draw a ticket allocating them to either GERD-education or control groups. If "no" the procedure would be postponed until a scheduled time was found suitable. The allocations to either GERD-education or control groups, maximum 8 patients in each group, would go on until either all allocations were filled, or until the teaching session started.

### GERD – education

A GERD – education program was developed for the purpose of this investigation by the main researcher (JU) and reviewed by a specialist in gastroenterology (HP) and a general practitioner, the latter two both holding senior academic positions. The program was conducted in groups to take advantage of group dynamics, stimulate each patient to participate in discussions and dialogue, and to lower the costs per patient as compared to individual consultations. The program was structured as three lessons that took place in the evenings spaced over two weeks. Each lesson lasted two hours. The content and structure of the program was explained on the first lesson. The first lesson conveyed basic information about GERD symptoms and pathophysiology, the second about pharmacological and non-pharmacological treatment and the third lesson about use of health services, communication and patients' rights. The essences of the previous lessons were repeated on the second and third lessons. The education program was designed as a structured dialogue allowing the patients to comment, ask questions and relate their own experiences to the information given on over-head transparencies. The patients were encouraged to disclose and discuss their worries, experiences and ideas in the group. All patients in the education group were given copies of the transparencies. Patients who did not attend all lessons were given an oral presentation of the information they missed either by telephone or by an extra class. The program was led by a medical doctor trained in educational counselling (JU) and was tested in a pilot study. After having attended the program, the GERD-education group was to be followed up by their general practitioner as part of routine care in the same manner as the control group.

### Statistical analysis

Patients who responded to both knowledge tests and questionnaires in the follow-up were classified as completing the study per protocol. In addition, patients in the education group were required to have attended at least one lesson to be classified as completing per protocol. Analyses were conducted on an intention-to-treat basis with serial mean imputation of missing items and results are reported as such. In addition per protocol analyses were performed but showed no significantly different results and are not reported.

Differences in GERD-knowledge were tested using student's t-test for independent or related groups as appropriate.

Associations between GERD-knowledge and socio-demographic variables, disease history, disease characteristics, DSIQ-ss and GHQ-lss at baseline were analysed in bivariate correlations. Variables correlating at a statistical level of p < 0.20 were selected as independent variables for stepwise multiple linear regression analysis with GERD-knowledge as the dependent variable.

Finally, the association between GERD-knowledge change from 2 to 12 months, and change in DSIQ and GHQ-30 profile and sum scores (QoL variables) were analysed in multiple linear regression models adjusting for selected covariates. Potential covariates were first identified by correlating baseline variables with change in GERD-knowledge. Baseline variables correlating with p < 0.20 were entered as independent variables in stepwise multiple regression analysis with change in GERD-knowledge as dependent variable. Baseline variables remaining in the model with p < 0.05 were entered as covariates in the final adjusted regression models. The final adjusted regression models were thus established using change in GERD-knowledge as the dependent variable, and using change in the QoL variables as independent variables adjusting for the selected covariates. Separate analyses were performed for all patients, patients having completed primary school only and patients having completed advanced school. These separate analyses were also performed stratified by allocation (GERD-education and controls).

Correlations were performed by parametric and non-parametric analyses as appropriate.

Statistical significance was set to p < 0.05. SPSS ver. 14.00 was used for all calculations.

Based on a small pilot study a sample size of 200 patients was found to be obtainable within a reasonable time span, and was considered to be an adequate number for analysis.

### Ethics

The study was conducted according to the Declaration of Helsinki, and approved by the Regional Committee for Medical Ethics in Trondheim, and the Data Inspectorate Norway. Each patient was informed about the study and gave a written consent to participation.

## Results

### Patients

Of 211 patients who were included, 102 were randomly allocated to GERD education and 109 to control. The GERD -education and control groups did not differ statistically with regards to socio-demographic variables; disease history or disease characteristics (Table 1). Of the patients who were allocated to GERD -education, 58 (56.9%) attended three lessons, 27 (26.5%) two and 5 (4.9%) one lesson. Twelve patients (11.8%) did not meet at all, of whom 5 were not able due to work or private obligations, 4 gave no reasons, 2 on grounds of other diseases and 1 did not wish to participate. At two months follow-up 90 patients (88%) in the GERD -education group and 100 patients (92%) in the control group responded completely. At 12 months 85 (83%) and 88 patients (81%) gave complete responses respectively.

**Table 1:** Socio-demographic and disease characteristics of patients allocated to GERD-education vs. control groups, recorded at baseline.

	**Education (n = 102)**	**Control (n = 109)**
Age, years	47 (12)	47 (14)

Sex (female)	49%	50%

Primary school only	31%	34%

Cohabitating	77%	80%

Blue-collar worker	22%	22%

BMI (kg/m^2^)	26 (3.6)	26 (4.7)

Previous history of serious disease	14%	17%

Length of GERD history (months)	166 (151)	169 (154)

Esophagitis	60%	51%

Having used H2-blocker or PPI	72%	63%

Ranitidine responder	41% (n = 74)	47% (n = 71)

Somatic comorbidity	53%	54%

Psychiatric comorbidity	8%	10%

Continuous variables are means (SD), categorical variables are percentages.

### GERD-knowledge test

**Figure 1:**
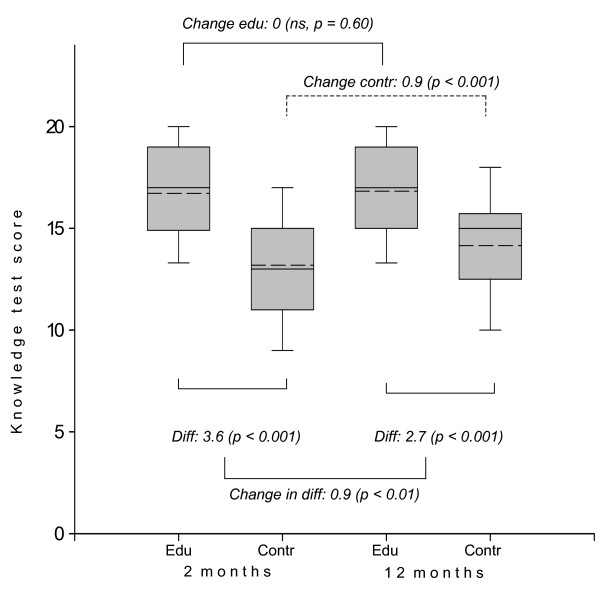
**Knowledge test scores at 2 and 12 months follow-up for educated patients (edu) and patients allocated to control (contr).** Plots show median (--), mean (- -), interquartile range (box) and 10th/90th (whiskers) percentiles. Diff. = difference in means.

Figure 1 shows mean GERD-knowledge at 2 and 12 months. The GERD -education group scored at both times higher than the control group (p < 0.001). The controls increased their knowledge test score significantly from 2 to 12 months follow-up (p < 0.001), whereas the GERD-educated patients did not. The difference in GERD-knowledge change between the patient education group and controls was 0.9 (p = 0.01).

GERD-knowledge correlated at a significance level of p < 0.2 with 8 socio-demographic variables, disease history or disease characteristic variables and GHQ-lss at baseline in addition to random allocation. Multiple linear regression analysis rendered GERD -education, having completed advanced school, a lower GHQ-lss and absence of psychiatric illness at baseline as independently associated with higher knowledge test scores. The highest beta-value and explained variance (R^2^) was found for allocation to GERD-education both at 2 and 12 months (Table 2). The length of GERD history showed no significant association to GERD-knowledge.

**Table 2:** Multiple linear regression analysis of knowledge test scores at 2 and 12 months follow-up as dependent variables and independent variables at baseline which correlated with knowledge test score at p < 0.2

	**2 months**			**12 months**		
*Independent variable *^*1*^	***Beta***	***p***	***R*^*2 *^*change***	***Beta***	***p***	***R*^*2 *^*change***

Allocation to education group	3.6	*< 0.001*	0.29	2.7	*< 0.001*	*0.17*
Formal educational level	1.1	*< 0.001*	0.06	1.2	*< 0.001*	*0.07*
GHQ-score at time of inclusion	- 0.1	*< 0.001*	0.08	- 0.2	*< 0.001*	*0.12*
Psychiatric disease at time of inclusion	- 1.6	*0.01*	0.02	- 1.5	*0.02*	*0.02*
R^2 ^(adjusted) for the model	0.44			0.36		

^1 ^Variables excluded in both models: Previous history of serious disease, esophagitis, having used PPI or H2-blocker, DSIQ sum-score, global QoL.

Allocation to GERD-education, a higher number of days on sick leave at the time of inclusion, and previously serious disease at baseline correlated negatively to an increase in GERD-knowledge from 2 to 12 months (p < 0.20), while total duration of GERD correlated positively. Variables selected in the stepwise regression analysis and entered in the final adjusted regression models were number of days on sick leave at the time of inclusion and allocation to intervention.

There was no statistically significant association between change in QoL variables and change in GERD-knowledge for the whole group of patients. An association was found between an increase in GERD-knowledge and deterioration in sense of coping (GHQ-30 profile: Coping failure) for patients who had completed advanced school (Table 3). For patients with primary school only, associations in the same direction were found in the DSIQ scales Gastric dysfunction, Life impairment and sum score, and an increase in GERD-knowledge test score. The explained variances in the final regression models (R^2^) were 0.1 or less. We found no major differences in these results when analyses were performed separately for the GERD-education and the control group.

**Table 3:** Associations between change in GERD-knowledge from 2 to 12 months follow-up and change in QoL-scores (DSIQ and GHQ-30 and their subscales).

	**Digestive Symptoms and Impact Questionnaire – DSIQ**						**General Health Questionnaire v. 30 – GHQ-30**					
	***Pain & bowel s.***	***Gastric dysfunc.***	***Health impair.***	***Life impair.***	***Reflux***	***Sum score***	***Anxiety***	***Well-being***	***Depr.***	***Coping***	***Social dysfunc.***	***Likert sum sc***

**All patients**	0.2 (0.18)	0.2 (0.18)	0.1 (0.59)	0.3 (0.10)	0.02 (0.84)	0.3 (0.13)	0.44 (0.25)	0.38 (0.46)	0.30 (0.50)	0.8 (0.06)	0.2 (0.60)	0.03 (0.15)

**Advanced school**	0.1 (0.65)	0.0 (1.0)	0.2 (0.51)	0.1 (0.66)	0.05 (0.76)	0.02 (0.57)	0.6 (0.16)	- 0.8 (0.22)	0.8 (0.22)	**1.0 (0.05)**	0.2 (0.49)	0.04 (0.08)

**Primary school only**	0.5 (0.09)	**0.7 (0.01)**	0.07 (0.81)	**0.8 (0.01)**	- 0.01 (0.95)	**0.8 (0.04)**	- 0.04 (0.96)	- 0.43 (0.63)	- 0.2 (0.79)	0.2 (0.81)	- 0.04 (0.95)	0.0 (0.92)

Numbers are β-values (p) in linear regression analyses adjusted for selected baseline variables (see text) with change in GERD-knowledge as dependent variable. Positive numbers indicate a reduced quality of life with increasing GERD-knowledge. β-values (p < 0.05) are in bold.

## Discussion

Patients with mild GERD to be followed up by their general practitioner after having been to gastroscopy, who attended a dialogue and group-based education program increased their disease knowledge as compared to GERD-patients in routine care, and their knowledge was retained for 12 months.

Recommending lifestyle changes to GERD patients has been considered beneficial [20,21]. However, few studies have evaluated the effect of systematic, structured patient education and to our knowledge there are no randomised controlled trials like ours relating patient education, disease knowledge and quality of life in patients with GERD.

In our study the strongest relationship was found between GERD-knowledge and being allocated to the GERD-education program. However, knowledge might be also be influenced by disease characteristics and socio-demographic variables [12,22]. We found that self reported presence of psychiatric disease and poor QoL as shown by GHQ-lss at baseline inversely correlated to GERD-knowledge. GHQ-30 is sensitive to depressive states and anxiety and may actually recognise poor mental health which is not recognised by the patient as such. The patients in our study would not be expected to be seriously impaired by psychiatric disease as the inclusion criteria ruled out patients who had other diseases than GERD that affected quality of life. Our findings suggest that even barely recognisable, minor reductions in mental health are associated with reduced learning which eventually may affect clinical outcomes. Although not surprising, our findings do remind us that we must attend to patients' depressive moods also in GERD-education.

In "Patient Education – A Practical Approach" [23], Kate Lorig underlines the necessity of analysing what patients want and need when planning an education program, tailoring the program as patient groups change. Our study shows, not unexpectedly, that patients who have completed advanced school have a higher level of knowledge irrespective of allocation to education or control group. This may have several explanations: Firstly these patients may be more used to acquiring knowledge in general through information seeking and may also better understand the language of their doctor than those with less education. Secondly, our knowledge-test may simply favour patients who are used to such testing. Our findings thus support the need for tailoring patient education programs to the qualifications and premises of the participants.

There was a contradictory finding in our study: Although patients in the control group significantly increased their knowledge over one year, the length of GERD history did not predict knowledge test score. It is reasonable to explain the increase in GERD-knowledge of the control group with time by the learning effects of filling in the test and the learning motivation precipitated by participating in the study.

The presumption that increased GERD-knowledge in some way lead to an improved quality of life, e.g. through reinforcing the reassuring effect of gastroscopy [24] was not supported by our data – neither when analysed overall nor when stratified by allocation. The explained variance in the final regression models were small (R^2 ^< 0.1) and we suspect the few associations we found between increase in GERD-knowledge and change in quality of life to be spurious. We believe this indicates that the positive effect of patient education on QoL in patients with primary school only [9] is not mediated through increasing their level of disease knowledge, but perhaps through deeper insight into already available information which relates GERD-knowledge more adequately to attitudes and skills.

Two major impressions of group responses were referred to by the by-sitter (HP) and the educator (JU) when the participants were encouraged to disclose worries and disease experiences: Participants expressed relief when realizing their experience of symptoms was shared with others in the group and pleasure in sharing ideas on how to manage symptoms.

The educational method applied in our study was "structured dialogue", inspired by the South American pedagogue Paulo Freires "liberation pedagogy". In practice this means introducing medical information while checking out if the patients recognise this within their own "universe of experience". This method has elements of cognitive behavioural therapy – as cognitive behavioural therapy has educational elements. Further research is needed to explore these comparative procedures.

Some critical points of the study need to be commented on: Our major outcome variable, GERD-knowledge, was not recorded at baseline. The reason for this was two-fold: 1) a knowledge test was by itself considered to be educational, which opposed the notion of the control group as one which was "treated as usual". 2) By random allocation we felt justified to assume that variables of importance for learning and level of preintervention knowledge would be equal in the two groups, thus a post intervention comparison would represent the effect of the education program.

Our results may not be valid for patients with severe GERD. Patients with severe GERD tend to be followed up by gastroenterologists at the hospital and thus receive other information that could influence upon the outcome. Furthermore, the study population, patients with mild GERD, might possess a "ceiling effect", i.e. the impact of disease might actually be too small to allow any intervention to lead to clinically and statistically significant improvement. Another weakness of our study was our knowledge test which was constructed by medical doctors paying attention to factual knowledge of medical relevance. Lorig et al [23]M:/GAR/REFS/felles.ref #334; points out the importance of patient involvement when defining learning goals in patient education. We recognise that a future knowledge test perhaps should be constructed in close collaboration with experienced patients defining goals relevant to living with the disease. However, other disease related knowledge tests have been constructed in the same manner [11] and the knowledge themes we tested were agreed upon by experienced doctors and therefore represented information which presumably would be conveyed within doctor-patient consultations. If so, our study shows that our GERD-education program supersedes patient information given within the usual consultation context.

## Conclusion

The group based education program in patients with GERD increased disease related knowledge. Patients with primary school only, minor psychiatric disease and a low QoL achieved a lower knowledge level and deserve special attention in future educational programs. Change in knowledge was not associated with change in quality of life which indicates a complex association between the two which needs to be further explored.

## Appendix

Knowledge test for patients concerning gastroesophageal reflux disease: Statement – and its correct characteristic as true or false.

1. Reflux disease is a rare disease – false.

2. Bloating is a symptom in reflux disease – false.

3. Cough may be a symptom of reflux disease – true.

4. Difficulties with swallowing may occur in reflux disease – true.

5 – 8: Which of the following occurs during an episode of reflux?

5. – The sphincter muscle between the stomach and the esophagus relaxes – true.

6. – The esophagus tightens – false.

7. – Acid leaks from the stomach into the esophagus – true.

8. – The production of bile increases – false.

9. Small meals will often increase reflux – false.

10. Fatty foods will often increase reflux – true.

11. Coffee will often aggravate reflux – true.

12. Late night meals may stimulate reflux – true

13. Bending forwards may worsen reflux – true

14. Nervousness is a cause of reflux disease – false.

15. Sedatives are an important treatment in reflux disease – false.

16. Medicine stimulating intestinal motility is used against reflux disease – true.

17. Acid production inhibitors are used against reflux disease – true.

18. Reflux may inflict ulcer in the esophagus – true.

19. Reflux may inflict ulcer in the stomach – false.

20. Constriction of the esophagus may occur as a consequence of reflux – true.

21. Reflux disease may lead to heart disease – false.

22. Blood tests may be used to prove a diagnosis of reflux – false.

23. Gastroscopy is an important investigation in reflux disease – true.

24. If in doubt one can measure the acidity in the stomach to clarify a diagnosis of reflux disease – false.

## Competing interests

The study was funded by an unrestricted grant from Glaxo Smith Kline, Norway, and supported by the Central Regional Health Authority and the Norwegian Fund for Health and Rehabilitation.

## Authors' contributions

JU conducted the study, performed statistical analyses and wrote the manuscript. HP initiated the study, reviewed patient referrals and gastroscopy reports for patient inclusion and contributed through all stages as senior researcher. PF contributed as senior researcher in concluding the study and writing the manuscript.

## Pre-publication history

The pre-publication history for this paper can be accessed here:


